# An Atypical Presentation of a Pediatric Mature Teratoma: A Case Report and Review of the Literature

**DOI:** 10.7759/cureus.72746

**Published:** 2024-10-31

**Authors:** Ahmed M Othman, Abdulaziz A Abu Alnasr, Reem E Kordi, Shahad A Abu Alnasr

**Affiliations:** 1 Emergency Department, Prince Mohammed Bin Abdulaziz Hospital, Madinah, SAU; 2 Family Medicine Department, Prince Mohammed Bin Abdulaziz National Guard Hospital, Madinah, SAU; 3 Pediatric Department, Prince Mohammed Bin Abdulaziz National Guard Hospital, Madinah, SAU; 4 Pediatric Hematology/Oncology Department, King Saud Medical City, Riyadh, SAU

**Keywords:** dermoid, mature cyst, ovarian, pediatric, saudi, teratoma

## Abstract

Immature teratoma is an uncommon type of malignant tumor that mostly consists of immature tissue originating from the neuroepithelium. Mature teratomas in pediatrics are relatively rare and difficult to diagnose because of various symptoms and the tendency to overlap with other illnesses. This study aimed to report a rare case of a mature teratoma in pediatric age. A Saudi female patient, aged 31 months, was brought to the emergency room (ER) in 2024 with a suprapubic mass. She did not complain of pain or urinary symptoms and had no substantial medical or family history, and the mass was discovered incidentally. A non-tender suprapubic mass was found during a physical examination. A large cystic mass containing fat and calcification was seen during imaging tests (pelvic CT and MRI), and this diagnosis was consistent with the presence of an ovarian dermoid cyst. The surgical intervention involved laparotomy with right oophorectomy, and the tumor was identified by pathological investigation as a mature teratoma, distinguished by a cystic structure packed with hair, gelatinous material, and bone components. Following the surgery, there were no issues, and a follow-up appointment was scheduled to monitor any complications. This case emphasizes the rarity and difficulties in diagnosing mature teratomas in pediatric patients, with positive and promising outcomes highlighting that a comprehensive diagnostic approach and a customized treatment plan are essential for a smooth recovery from ovarian lesions in pediatrics. Consequently, accurate diagnosis and quick surgical intervention are critical for optimal management, even in the absence of obvious symptoms.

## Introduction

The word "teratoma" comes from the Greek word "teraton," which means monster, and refers to the complicated tissue composition of the tumor [1]. There are three different types of teratomas: monodermal, mature, and immature. A malignant germ cell tumor known as an immature teratoma is distinguished by the presence of tissues originating from the ectoderm, endoderm, and mesoderm germ cell layers. It is often an immature tissue composed mostly of neuroepithelial cells [2]. An immature tumor is the second most prevalent malignant germ cell tumor after dysgerminoma, typically affecting adolescents and young adults of reproductive age [3]. It has an incidence of 2.3 per 100,000 patients. Immature teratomas constitute less than 1% of ovarian malignancies and 35.6% of all germ tumors in young women [4,5]. Mature teratomas or dermoid cysts are benign tumors that account for 95% of all teratomas. The disease is characterized by multiple differentiated tissue types, which range in diameter from 7 to 15 cm, and show components of the ectoderm, endoderm, and mesoderm layers [6]. The condition is characterized by unilaterality, cyst formation, infrequent septation, and sporadic manifestation of the Rokitansky nodule. It contains bone tissue, teeth, hair sebum, and fatty and keratin tissues [7]. The diagnostic process of ovarian tumors in young children is considered a challenging task due to their varied etiology and the potential overlap of their symptoms with other medical diseases [8]. A common benign ovarian tumor that can cause vague or non-specific symptoms is called a dermoid cyst or a mature cystic teratoma [9]. About 3% of ovarian tumors are mature teratomas and are found mostly in young girls [10]. It is a spherical, rubbery mass beneath the skin that develops during prenatal development. Similar to all skin cells, these cells proliferate, divide, and finally form into a ball of cells that produce sweat, hair, and proteins [11]. Following birth, the dermoid cyst keeps expanding and forming a benign tumor. However, mature tumors are dangerous because they can harm the surrounding bones and tissues [12]. While young girls with ovarian lesions are treated with surgery, treatment of adult patients routinely uses adjuvant chemotherapy for all cases, except those with grade 1 stage IA tumors [13].

Limited information is available regarding the diagnosis and treatment of mature teratomas in pediatrics due to their rarity. This case report describes mature teratomas of the ovary that were unintentionally discovered in a 31-month-old girl who presented to the emergency room with a suprapubic tumor.

## Case presentation

In 2024, a Saudi 31-month-old female patient presented to the emergency room (ER) with an incidentally discovered suprapubic mass, noticed incidentally by her mother during a diaper change three days earlier. She was asymptotic with no pain and no urinary symptoms such as a change in the color, amount, or smell of the urine. She had no fever, weight change, night sweats, vomiting, change in bowel habits, joint pain, skin rashes, or changes in consciousness or activity levels. Her past medical history contained a neonatal intensive care unit admission at birth due to respiratory distress that was treated with continuous positive airway pressure. Her past surgical history was unremarkable. No family history of malignancy or hematological disease was detected.

During the physical examination, she appeared well, afebrile, and vitally stable with no signs of respiratory distress. She was not dysmorphic or pale and had a capillary refill of less than 2 seconds and normal heart sounds. She weighed 12.5 kg and had a height of 97 cm. Her vital signs were a temperature of 36.7°C, respiratory rate of 30/minute, respiratory rate of 120/minute, blood pressure of 94/47 mmHg, and saturation of peripheral oxygen of 98%. There was no throat congestion or tonsillar hypertrophy, and her tympanic membranes were not inflamed. There was normal and stable air entry to the chest and good peripheral pulses. The abdominal examination revealed a soft and lax abdomen with a 4×4 cm round, non-mobile, non-tender, cystic suprapubic mass, resonant in percussion, without lymphadenopathy or organomegaly. Neurologically, she had a Glasgow Coma Scale (GCS) score of 15/15. Her pupils were round, equal, and reactive. She had normal muscle power and reflexes.

**Figure 1:**
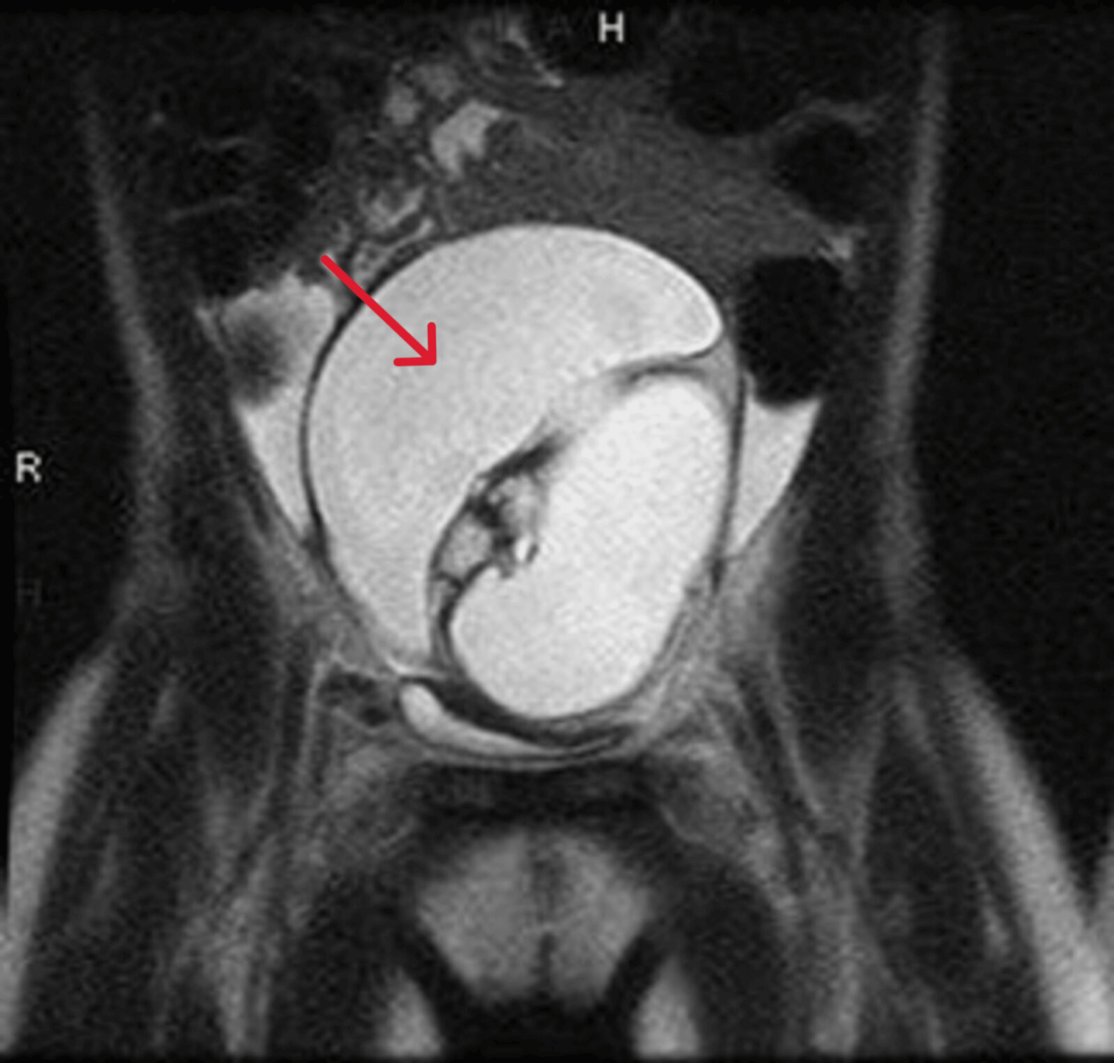
MRI report revealing a large complex cystic lesion in the pelvis, likely a mature cystic teratoma, measuring about 5.7×7.1×8.9 cm (arrow) The right ovary is not visualized, while the left ovary contains normal follicles. The uterus appears normal, and there is a small amount of free fluid noted. No lymphadenopathy or bowel abnormalities are present.

**Figure 2:**
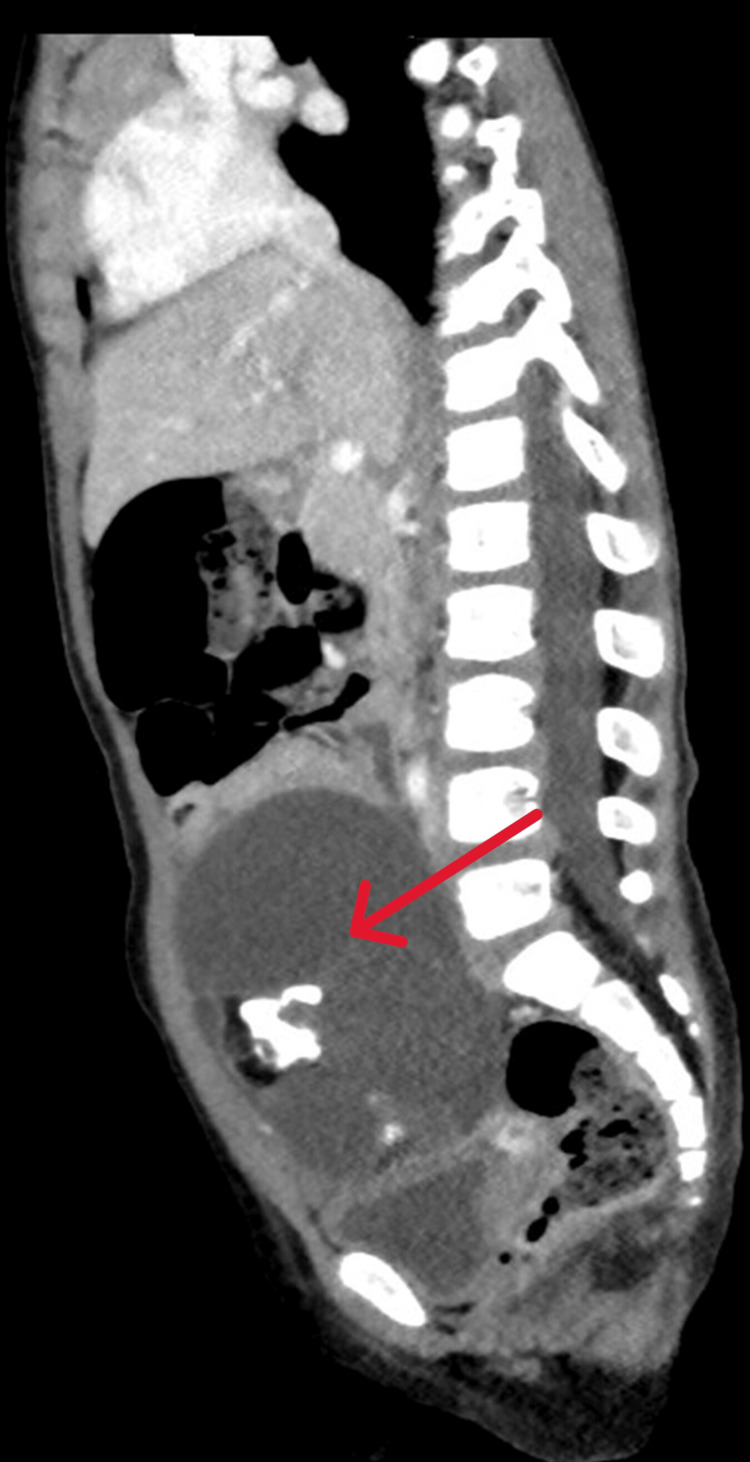
Imaging findings revealing a central cystic pelvic mass measuring 7×6.3×8.4 cm, consistent with an ovarian dermoid, causing compression on the urinary bladder (arrow) There is minimal free pelvic fluid, but all other abdominal organs, including the liver, gallbladder, pancreas, spleen, kidneys, and adrenal glands, appear normal with no lesions. No significant lymphadenopathy or free fluid is noted in the abdominopelvic cavity, and the gastrointestinal tract is unremarkable. The ovarian dermoid is likely left-sided, although its exact origin is uncertain.

As mentioned, Figure 1 and Figure 2 present the results of the imaging studies, including pelvic CT and pelvic MRI. Both studies identified a large cystic pelvic mass with fat and calcification. In Figure 2, the pelvic CT further reveals minimal free pelvic fluid and confirms normal findings for the liver, gallbladder, pancreas, spleen, kidneys, and adrenal glands. Additionally, there is no evidence of mesenteric or retroperitoneal lymphadenopathy.

**Figure 3:**
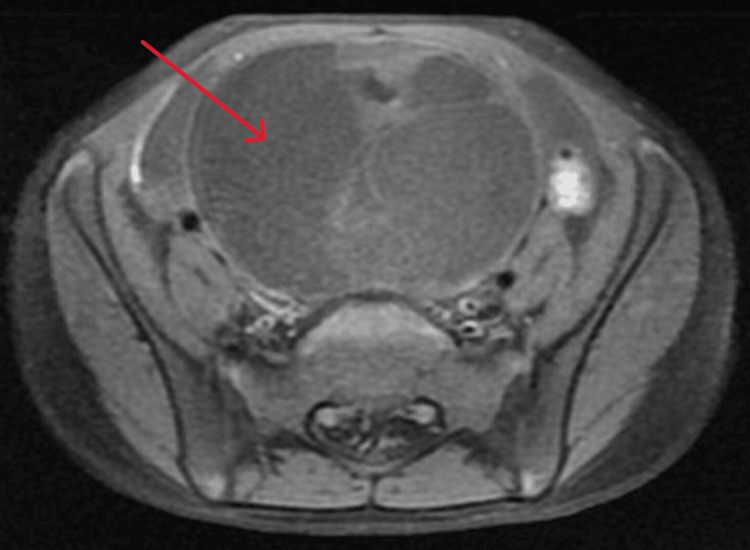
MRI findings indicating a large midline pelvic complex cystic lesion containing fat and calcification, most likely a mature cystic teratoma arising from the right ovary (arrow) The left ovary shows follicles, likely due to hormonal effects. There are no abnormalities noted in the bones or other significant findings.

In Figure 3, the pelvic MRI identified a large midline pelvic cystic lesion measuring 5.7×7.1×8.9 cm, containing fat and calcification, with multiple enhancing septations, which is compressing the bladder. The uterus appeared normal, and the left ovary appeared with follicles, which is likely secondary to hormone effects. The cytology report indicated that the peritoneal fluid was non-diagnostic due to its inadequacy as it contained blood only. The patient went under laparotomy with a right oophorectomy.

The surgical pathology report showed that the right ovarian mass consisted of a cystic structure measuring 8.5×7.0×6.0 cm. The outer surface was gray-tan smooth and glistening. It weighed 222 g. There was an attached tubular structure of the cyst measuring 5.5×0.3 cm. Serial sectioning revealed a multi-locular cyst filled with hair tuft, cheesy material, and bony structures. No residual ovarian tissue was identified. Post-operative recovery was uneventful, and the patient was discharged with a follow-up planned to monitor for any potential recurrence or complications. Upon discharge, the patient was referred to the Pediatric Surgery Department at Prince Mohammed bin Abdulaziz Hospital, where pediatric surgeons recommended continued follow-up for further evaluation at King Abdullah Specialized National Guard Children's Hospital in Riyadh. At this facility, the patient was assessed by a multidisciplinary team, and she was given a biannual follow-up plan with pelvic ultrasound to be re-repeated before each visit.

## Discussion

The clinical presentation and epidemiology of ovarian lesions in pediatric patients vary globally. In general, its incidence is uncommon, and it is frequently found incidentally. A mass in a 31-month-old girl who was addressed in this study was discovered incidentally during an ER visit, and the girl was diagnosed with an asymptomatic suprapubic tumor. A large cystic mass compatible with an ovarian dermoid cyst was discovered by imaging techniques. After surgical resection, the mass was specified as mature teratoma, with characteristic hair, cheesy substance, and bony structures. After surgery, the patient recovered completely without any serious consequences. This example is particularly noteworthy because young children rarely develop this disease.

About 50% of ovarian tumors in children and 20% of ovarian tumors in adults are mature cystic teratomas. Bilaterality is prevalent 8%-15% [10]. Occasionally, as reported in this study, the identification of those tumors is typically coincidental during a normal abdominopelvic ultrasound examination, when an abdominopelvic mass is palpated. According to previous studies, 16% of instances of this lesion may be exacerbated by torsion, 2% by malignant degeneration, 1.2%-3.8% by rupture, and 1% by infection [14].

Overall, the examination with ultrasound showed a cyst with multiple linear echoes that correspond to hairs floating in the cyst lumen or a cyst with an echogenic parietal nodule that corresponds to the Rokitansky nodule or protuberance. Other forms may be recorded such as a common cyst with a serous content or a cyst with a liquid level with a serous liquid and supernatant sebum [15]. As demonstrated by both the CT and MRI results, the case study is consistent with the typical presentation of a mature teratoma, which is typically unilateral, cystic, and frequently accompanied by fat and calcification. The present case is in line with another case study of an eight-day-old female newborn suffering from mature ovarian teratoma. The abdominal ultrasound was ambiguous and suggested the possibility of neuroblastoma, a condition that frequently manifests with an abdominal mass in newborns [16]. Due to the precision of CT scans in visualizing various components of tissue, fat, fluid, and calcium, it is considered the greatest diagnostic tool available. On the other hand, the diagnosis made by MRI can show the exact percentage of fat.

A retrospective chart review of all cases that included pediatric ovarian lesions at King Abdulaziz Medical City in Riyadh, Saudi Arabia, between January 1997 and August 2016 revealed 14 cases [17]. Approximately 35.7% of the patients received a diagnosis after presenting with severe abdominal pain, whereas 28.6% of patients received a diagnosis by chance without exhibiting any symptoms. According to the study, more than half of the patients (57.1%) had laparoscopies, and 50% of them had cystectomies. The present case was consistent with the previously mentioned study in the fact that a suprapubic mass was incidentally discovered. It differed from the same study in that the patient had laparotomy with right oophorectomy after imaging studies identified a large cystic mass in the pelvis consistent with a dermoid ovarian cyst. The size of the mass (7×6.3×8.4 cm) and its compressive effect on the bladder, along with the non-diagnostic peritoneal fluid, made laparotomy and right oophorectomy necessary.

Ovarian lesions in pediatric patients have been subjected in multiple cases. Fifty-eight children with ovarian lesions aged between nine and 15 years were reported in a study that was conducted at the University Department of Pediatric Surgery and Urology in Wroclaw, Poland, between 1991 and 2016. This age range corresponds to the typical range for ovarian teratomas in pediatric patients [18]. According to this study, 23 (41.82%) and 28 (50.91%) of the females with mature teratomas received laparoscopies and laparotomies, respectively. In comparison, our patient at 31 months was significantly younger, reflecting the younger age range where teratomas are less commonly diagnosed. This discrepancy highlights the variability in age at presentation. Laparotomy or laparoscopy was used to treat the majority of mature teratomas, demonstrating the difference between minimally invasive and conventional surgical techniques. The huge size and compressive impact of the mass in our case necessitated a laparotomy, supporting the study's conclusion that more intrusive surgical methods are frequently required for larger or more complex tumors. Adjuvant treatment after a formal laparotomy was necessary for advanced stages of all cases of immature teratomas in the abovementioned study. This approach emphasizes how aggressive care is necessary in cases of malignancy. Given the benign nature of the mature tumor in our case, no adjuvant treatment was required.

Differential diagnosis becomes difficult when ovarian tumors in pediatric patients are asymptomatic or have nebulous symptoms. The patient in our case was asymptomatic and exhibited a suprapubic tumor that was unintentionally found during an ER visit. This is consistent with the research that suggests ovarian tumors, including teratomas, may not always exhibit obvious clinical symptoms and may be misdiagnosed as benign illnesses [19]. Because mature cystic teratomas are mobile within the adnexal structures and have a cystic character, they are particularly linked to adnexal torsion. It has been noted that mature teratomas might produce torsion; however, neither rupture nor torsion was seen in our case. After the surgery, the patient had a non-tender mass without any acute consequences, and the imaging studies did not indicate any evidence of torsion. This is consistent with research showing that malignant ovarian tumors are less likely to twist because of their propensity to stick to nearby structures, a characteristic associated with desmoplastic reactions [18].

## Conclusions

This case study highlights the diagnostic and therapeutic issues of ovarian dermoid cysts in pediatrics. An incident involving the discovery of a large, asymptomatic suprapubic tumor in a 31-month-old girl underscores the vital role that imaging techniques play in identifying and distinguishing mature teratomas from other conditions. Although adult teratomas are often benign, effective care requires surgery due to the mass's large size and compressive impact on nearby structures. Timely surgical intervention and comprehensive follow-up are crucial, as evidenced by the patient's favorable prognosis and seamless recuperation. The present case, together with other relevant reviews, highlights the need for personalized treatment approaches and ongoing monitoring to ensure the best possible care for pediatric ovarian lesions. In order to improve outcomes and resolve the challenges posed by uncommon pediatric tumors such as ovarian dermoid cysts, future research should focus on improving diagnostic techniques and treatment regimens.
